# Climatizing internal medicine training: design, implementation, and evaluation of an environmental health curriculum for residents

**DOI:** 10.3389/fpubh.2026.1820889

**Published:** 2026-04-29

**Authors:** Jessica Y. Chambers

**Affiliations:** Department of Internal Medicine, Dell Medical School, University of Texas at Austin, Austin, TX, United States

**Keywords:** climate change, environmental health, environmental justice, graduate medical education, internal medicine, medical education, residency curriculum, self-efficacy

## Abstract

**Background:**

Climate change poses an escalating threat to human health, yet graduate medical education has been slow to incorporate environmental health content into residency training. Internal medicine residents report low confidence in their ability to discuss environmental health topics, creating gaps in patient counseling and health advocacy. Despite position statements from major medical societies calling for environmental justice education in graduate medical education, few published curricula exist to guide internal medicine residency programs in this effort.

**Objective:**

To describe the design, implementation, and preliminary evaluation of an interdisciplinary environmental health curriculum for internal medicine residents at a large university-affiliated residency program in the southern United States, informed by a formal needs assessment identifying specific knowledge gaps.

**Methods:**

A seven-session environmental health curriculum was developed using a needs assessment-driven approach grounded in Bandura’s self-efficacy theory and Kern’s six-step framework for curriculum development in medical education. Twelve environmental health topics were assessed via a baseline confidence survey of 110 internal medicine residents. Curriculum content was prioritized based on the lowest-confidence domains and delivered through interactive didactic sessions led by content-area experts across disciplines including pulmonology, infectious disease, toxicology, and public health. Sessions covered climate change and human health, environmental justice, food security, endocrine disruptors, air pollution, emerging infectious disease, extreme weather, climate and cancer, and occupational lung disease. Post-curricular confidence surveys were administered to evaluate impact.

**Results:**

The baseline needs assessment (*n* = 62, 56% response rate) revealed a mean confidence score of 2.22 out of 5 across all environmental health topics. Hazardous waste, endocrine disruptors, water quality, toxicology, and environmental justice represented the lowest confidence domains. Seventy-one percent of residents reported no or slight confidence discussing environmental justice with peers. The curriculum was successfully integrated into existing didactic time across seven sessions from April through December 2024, with positive resident engagement and satisfaction. Post-curricular assessment data analysis is ongoing.

**Conclusion:**

This article presents a replicable, needs assessment-driven model for embedding environmental health education into internal medicine residency training. By grounding curricular design in self-efficacy theory and leveraging interdisciplinary expertise, programs can systematically address the gap between the growing health impacts of climate change and physician preparedness to counsel patients and advocate for communities.

## 1 Introduction: background and rationale

The health consequences of climate change are clinical realities confronting physicians today. Heat-related morbidity, air pollution-associated respiratory disease, vector-borne illness expansion, and the mental health sequelae of extreme weather events are increasingly presenting across clinical settings (1–3). Vulnerable and marginalized communities bear a disproportionate burden of these hazards, deepening health inequities through mechanisms rooted in structural racism and socioeconomic disadvantage (4, 5).

Despite the growing clinical relevance of these issues, graduate medical education (GME) has been slow to integrate environmental health content into training programs. A 2024 survey of family medicine residency program directors found that over 56% of programs had no climate change-related curricular content and no plans to introduce it (6). A scoping review of climate change curricula in U.S. GME programs published in 2024 similarly found very few published accounts of climate-related education in residency training, with existing efforts concentrated largely in pediatrics and emergency medicine (7). Internal medicine, the specialty responsible for the broadest swath of adult patient care, has been notably underrepresented in this emerging educational landscape.

Multiple medical societies have issued calls to address this gap. The American College of Physicians published a position paper in 2022 urging curricular standards for environmental health in medical training (8). The American Medical Association has endorsed the inclusion of climate change and health content across the continuum of medical education (9). Philipsborn et al. proposed the first residency curriculum framework linking climate and health learning objectives to Accreditation Council for Graduate Medical Education (ACGME) core competencies (10). Sullivan et al. described an integrative model for weaving climate and health content into established medical school curricula without displacing traditional biomedical material (11). Yet, translating these frameworks into implemented, evaluated curricula within internal medicine residency programs remains an unmet need.

A critical first step in curricular design is understanding where trainees currently stand. We previously published a needs assessment of internal medicine residents’ self-perceived confidence in explaining environmental health topics, which revealed uniformly low confidence across 12 domains (12). These findings provided the empirical foundation for the curriculum described in this article.

The present article describes the design, implementation, and preliminary evaluation of an interdisciplinary environmental health curriculum developed for internal medicine residents at a large university-affiliated program in the southern United States.

## 2 Pedagogical framework

### 2.1 Bandura’s self-efficacy theory

The theoretical foundation of this curriculum is rooted in Bandura’s social cognitive theory, specifically the construct of self-efficacy—an individual’s belief in their capacity to execute behaviors necessary to achieve specific outcomes (13). Self-efficacy is not merely confidence in the abstract; it shapes whether individuals attempt a task, how much effort they invest, and how they persist in the face of difficulty (14). In medical education, self-efficacy has been linked to clinical performance, communication skills, and the likelihood that trainees will engage in patient counseling on complex topics (15, 16).

Bandura identified four primary sources of self-efficacy: mastery experiences (successful task performance), vicarious experiences (observing peers or role models succeed), verbal persuasion (encouragement and expert feedback), and physiological and affective states (managing anxiety and building comfort) (13). Our curriculum was designed to leverage each of these sources. Interactive didactic sessions with case-based discussion provide opportunities for mastery. Expert-led lectures by faculty from diverse specialties serve as vicarious learning experiences. Small-group dialogue and faculty mentorship offer verbal persuasion. Finally, the progressive, modular structure of the curriculum allows residents to build comfort incrementally across sessions, addressing the affective dimension of self-efficacy.

We applied self-efficacy theory specifically because our needs assessment measured confidence rather than knowledge per se. As Artino noted, self-efficacy beliefs are powerful mediators between knowledge acquisition and behavioral performance in clinical settings (14). A resident who understands air pollution pathophysiology but lacks confidence in translating that knowledge to patient counseling may never initiate the conversation. Therefore, our curricular design intentionally targeted confidence-building as the proximal outcome, with the expectation that improved self-efficacy would facilitate downstream improvements in patient education and health advocacy.

### 2.2 Kern’s six-step approach to curriculum development

The overall curricular development process followed Kern’s six-step approach, a widely adopted framework in medical education: (1) problem identification and general needs assessment, (2) targeted needs assessment, (3) goals and objectives, (4) educational strategies, (5) implementation, and (6) evaluation and feedback (17). This framework provided a systematic structure for moving from the identification of a training gap to the delivery and assessment of a tailored educational intervention. Each step is described in subsequent sections of this article.

### 2.3 Alignment with ACGME core competencies

**Figure 1:**
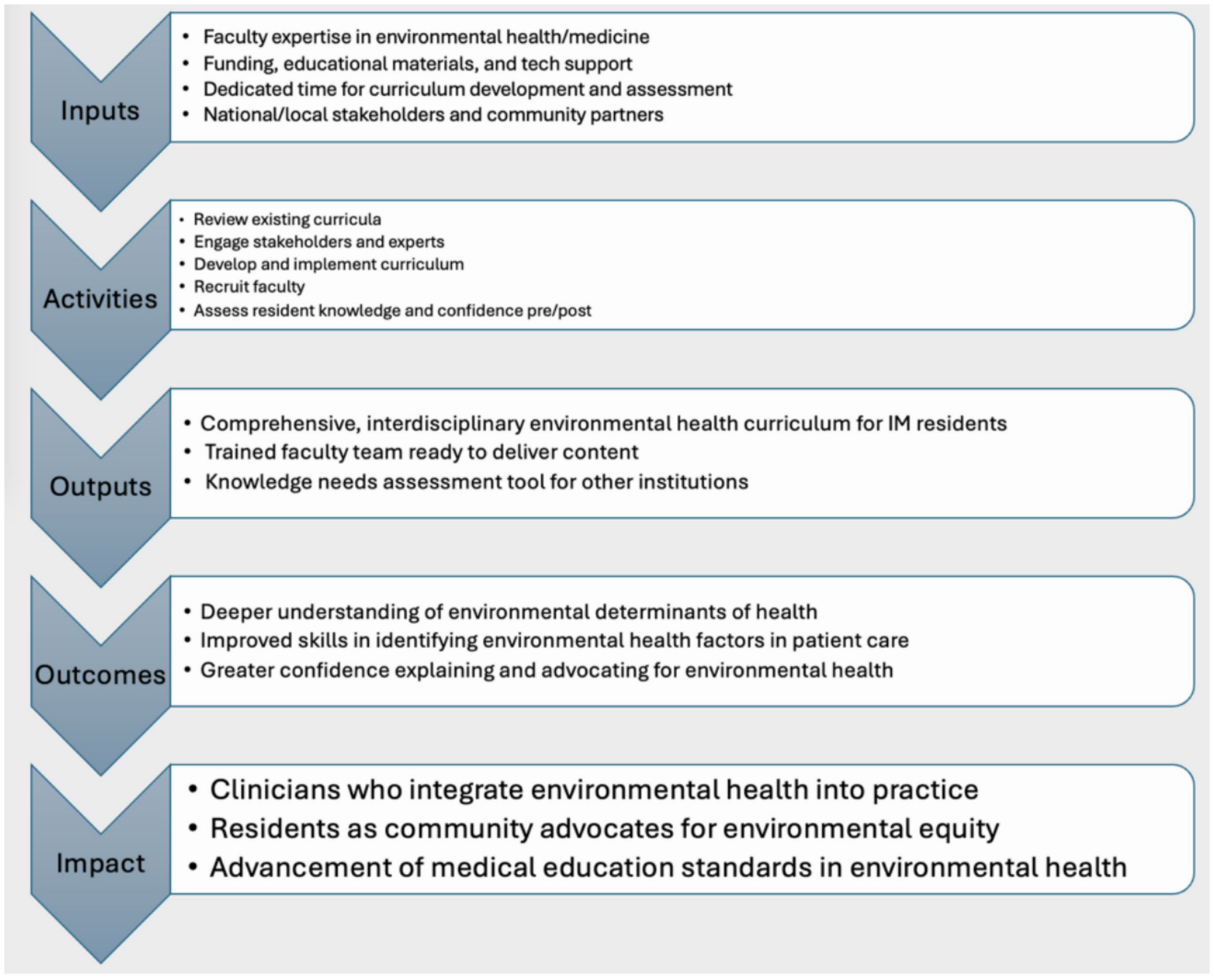
Environmental health curriculum logic model.

Although the ACGME has not yet incorporated climate-specific competencies into its Common Program Requirements, the environmental health topics addressed in this curriculum align with several established ACGME domains. Medical knowledge is advanced through evidence-based content on climate-health pathways. Patient care and procedural skills are supported by training residents to incorporate environmental exposure histories into clinical assessments. Systems-based practice is addressed through discussions of health system sustainability, environmental justice, and population-level impacts. Practice-based learning and improvement is fostered through reflective exercises and post-session assessments. Finally, the curriculum’s emphasis on health advocacy and community engagement supports interpersonal and communication skills as well as professionalism (10, 18). Figure 1 presents the logic model underlying the curriculum, illustrating the relationship between program inputs, educational activities, short-term outputs, and intended long-term outcomes.

## 3 Learning environment, objectives, and pedagogical format

### 3.1 Setting and participants

The curriculum was implemented at the University of Texas at Austin Dell Medical School, a university-affiliated internal medicine residency program in Austin, Texas. The program comprises approximately 110 residents across postgraduate years (PGY) 1 through 3, including both categorical (*n* = 84) and preliminary (*n* = 26) tracks. Austin, located in central Texas, provides a particularly relevant regional context for environmental health education: the city experiences extreme heat events, worsening air quality, urban heat island effects, and environmental inequities that disproportionately affect low-income and minority communities in eastern Travis County. This geographic relevance was deliberately leveraged to ground curricular content in locally meaningful clinical scenarios.

### 3.2 Needs assessment and topic prioritization

**Figure 2:**
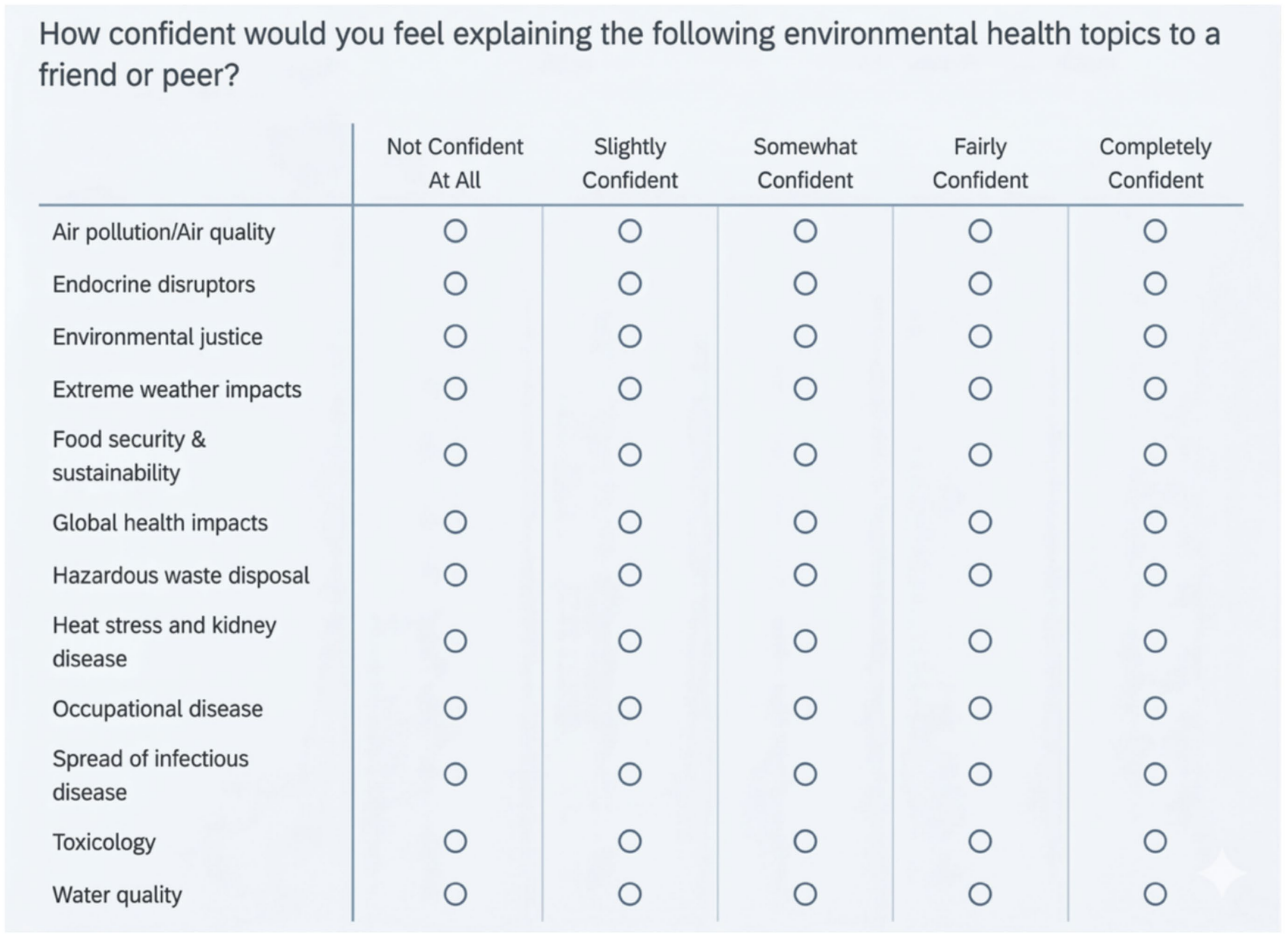
Environmental health topics needs assessment survey tool.

The curriculum was informed by a formal needs assessment conducted between February and July 2024. An online survey deployed via Qualtrics assessed residents’ self-perceived confidence in explaining 12 environmental health topics to a peer, using a 5-point Likert scale (1 = not confident at all to 5 = completely confident) (Figure 2). The survey instrument was developed through semi-structured interviews with clinicians and researchers in environmental health, refined via rapid qualitative analysis and expert review (12).

The needs assessment yielded a 56% response rate (62 of 110 residents). The overall mean confidence score was 2.22, corresponding to a level between “not confident at all” and “slightly confident.” The five lowest-scoring domains were hazardous waste disposal (mean = 1.73), endocrine disruptors (1.76), water quality (1.90), toxicology (2.02), and environmental justice (2.04). The highest-scoring domains were food security and sustainability (2.71) and spread of infectious disease (2.92), though even these scores reflect substantial room for improvement. Notably, 44% of respondents reported no confidence at all in discussing environmental justice, and 71% reported either no or slight confidence in this domain (12). These results directly informed the selection and sequencing of curricular modules.

### 3.3 Learning objectives

Each session was designed around specific, measurable learning objectives aligned with the environmental health topics identified in the needs assessment. Overarching curricular goals included the following: (a) examine the burden of environmental health disparities in central Texas and beyond; (b) apply foundational principles of environmental health science to patient evaluation and treatment plans; (c) understand how structural determinants produce uneven environmental exposures leading to health inequities; (d) identify vulnerable patient populations and develop individualized care plans addressing environmental risk factors; and (e) formulate strategies for patient education, community advocacy, and policy engagement related to environmental health.

### 3.4 Curricular content and session descriptions

Seven sessions were delivered between April and December 2024, each integrated into existing protected didactic time within the residency schedule. No existing didactic content was permanently displaced; sessions were scheduled during recurring protected educational time allocated for elective and special-topics lectures. The curriculum development process spanned approximately 6 months, from initial stakeholder interviews and needs assessment deployment (February 2024) through completion of lecture materials (April 2024). Estimated faculty preparation time was 15–20 h per session, including literature review, slide development, and case creation. Individual sessions were 60–90 min in duration. Sessions were led by faculty with content expertise spanning internal medicine, pulmonary and critical care medicine, infectious disease, toxicology, environmental science, and public health. The sessions and their focused topics were as follows. Each session followed a general structure of approximately 30–40 min of expert-led didactic presentation, 15–20 min of case-based small-group discussion, and 10–15 min of facilitated whole-group debrief and audience questions. Proportions varied by topic; for example, the infectious disease session (Session 5) was more heavily case-based with board-style questions, while the endocrine disruptors session (Session 3) allocated more time to didactic content given the technical nature of the material.

*Session* 1: Introduction to Climate Change and Human Health (April 2024). This foundational session introduced the relationship between climate change and health outcomes, with sub-topics on environmental justice and heat stress-induced chronic kidney disease. Objectives included examining environmental health disparities in central Texas, applying pollution inequality principles to clinical care, understanding how structural racism generates uneven environmental exposures, and identifying populations at risk for heat-related renal injury.

*Session* 2: Food Security and Sustainability (April 2024). This session examined the elements of food security, global patterns of undernutrition, and modeled projections of food insecurity under climate change scenarios through 2050. Sub-topics included global health impacts and extreme weather effects on food systems.

*Session* 3: Endocrine Disruptors and Toxicology (May 2024). Led by a senior scientist from a national environmental organization, this session defined endocrine disruptors, identified major chemical classes (bisphenols, per- and polyfluoroalkyl substances, phthalates, dioxins), explained dose–response relationships and threshold effects, and formulated exposure reduction strategies including patient education and policy advocacy.

*Session* 4: Air Pollution and Pulmonary Disease (May 2024). A pulmonary and critical care specialist led this session on the impact of particulate matter, nitrogen dioxide, and ozone on chronic obstructive pulmonary disease development, exacerbation, and healthcare utilization. Residents developed advocacy plans for improved environmental and occupational protections in collaboration with community organizers.

*Session* 5: Emerging Infectious Disease Due to Climate Change (May 2024). An infectious disease specialist presented board-style clinical cases illustrating emerging infections affected by climate change, including vector-borne and waterborne diseases, and described the mechanisms by which shifting temperature and precipitation patterns alter disease transmission.

*Session* 6: Extreme Weather Impacts and Heat Stress (October 2024). This session explored the intersection of extreme weather events and infectious disease (leptospirosis, cryptosporidiosis), urban runoff and carcinogenic exposure, the One Health concept, vulnerable populations in heat stress, and cold-related illness.

*Session* 7: Climate Change and Cancer/Occupational Lung Disease (November–December 2024). The final sessions addressed the disproportionate impacts of natural disasters on cancer care access, supply chain disruption effects on cancer treatment, environmental carcinogens (uranium, radon), allostatic load, occupational lung disease, volatile organic compounds, and occupational asthma.

### 3.5 Pedagogical format

All sessions employed an interactive didactic format, incorporating structured presentations interspersed with case-based discussions, small-group problem-solving exercises, audience response questions, and facilitated dialogue. This approach was selected to promote active learning and align with Bandura’s mastery experience and verbal persuasion sources of self-efficacy (13). The case-based components were designed to simulate clinical scenarios in which residents would need to counsel patients on environmental exposures, thereby bridging the gap between knowledge acquisition and clinical application.

**Figure 3:**
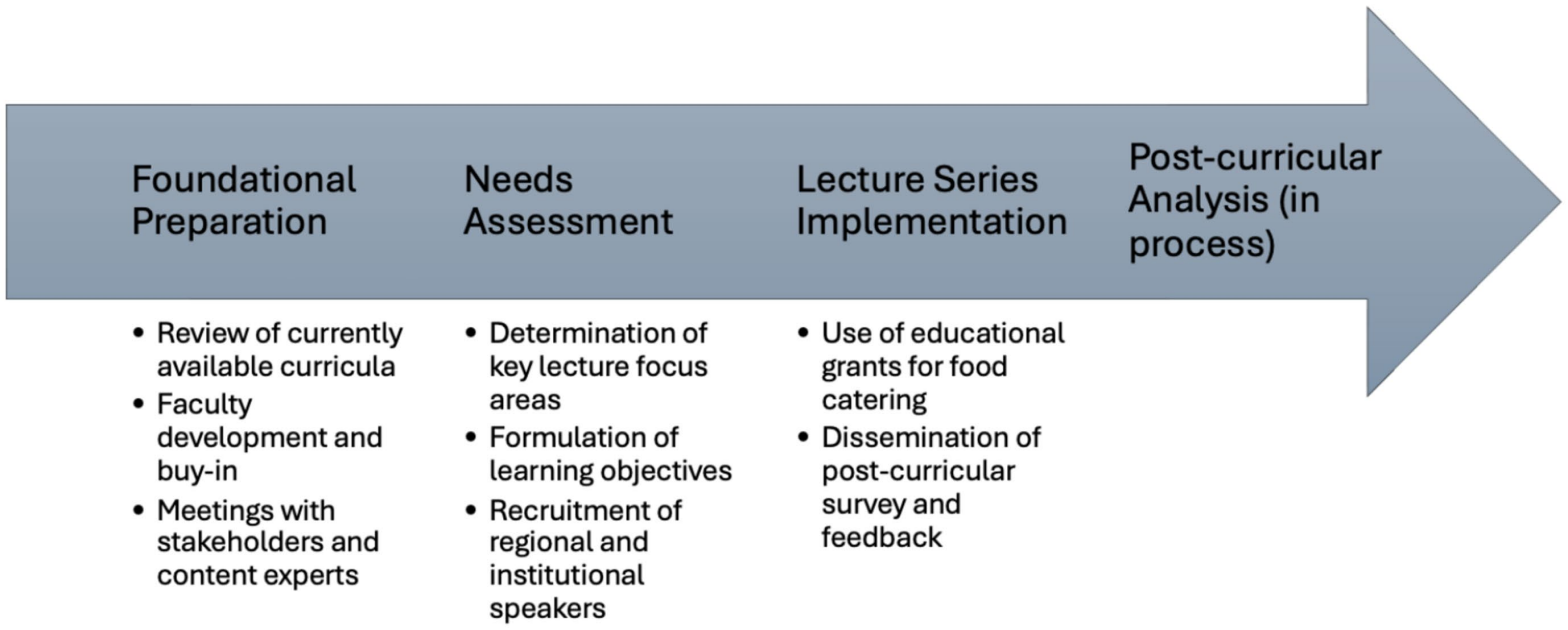
Flowchart of curricular design methods.

Faculty presenters were recruited for both content expertise and pedagogical engagement. Prior to delivery, each presenter received a curricular orientation document outlining the overarching learning goals, the self-efficacy framework, expectations for interactive pedagogy, and a standardized template for learning objectives. The author met individually with each presenter to review session content, ensure alignment with the needs assessment findings, and promote consistency across sessions. Despite these efforts, some variability in teaching style and depth of interactive engagement was observed across presenters, reflecting the inherent challenge of standardizing instruction across multiple disciplines. An interdisciplinary faculty team—including internists, pulmonologists, infectious disease specialists, toxicologists, and public health researchers—modeled the cross-disciplinary collaboration that environmental health practice demands. Sessions were catered with locally sourced food funded by educational grants, an intentional decision to create a welcoming learning environment and reinforce themes of food sustainability. A flowchart of the curricular design is available in Figure 3.

## 4 Results to date and assessment

### 4.1 Baseline assessment: the needs assessment

As described previously and published in detail elsewhere (12), the baseline needs assessment provided critical data guiding curriculum development. Among the 62 respondents, PGY-1 residents reported lower mean confidence than PGY-2/3 residents across most topics, with the exception of toxicology and environmental justice, where PGY-1 confidence was paradoxically higher. This finding may reflect increasing integration of social determinants of health and environmental themes into undergraduate medical education curricula in recent years (19). The statistically significant confidence gap between the five lowest-rated and two highest-rated topics confirmed that a targeted, tiered approach to curricular content was warranted.

### 4.2 Implementation outcomes

The seven-session curriculum was successfully integrated into the residency’s existing didactic schedule without requiring additional protected time. All sessions were delivered as planned between April and December 2024. Resident attendance was tracked through standard institutional processes. Anecdotal feedback from residents and faculty was positive, with residents reporting that the content was clinically relevant, engaging, and applicable to their daily practice. Several residents expressed interest in continuing environmental health advocacy activities beyond the formal curriculum, suggesting that the intervention may have activated a broader professional identity around health advocacy (20).

### 4.3 Post-curricular assessment

A post-curricular survey instrument was designed to mirror the baseline needs assessment, enabling direct comparison of confidence scores before and after curricular exposure. The survey was administered to all residents who attended at least one session. The post-curricular assessment employs the same Likert-scale confidence items as the baseline survey, allowing for pre-post analysis of mean confidence scores across each of the 12 environmental health domains. In addition, the post-curricular instrument includes knowledge-based items tied to specific session learning objectives; these items will be analyzed descriptively to assess factual recall and conceptual understanding. However, because the baseline survey did not include parallel knowledge-based questions, pre-post comparison is limited to the confidence domain. This limitation—the absence of a formal pre-intervention knowledge assessment—should be noted, and future iterations of the curriculum should incorporate matched knowledge items at both timepoints to enable more robust evaluation of learning outcomes. Data analysis is ongoing at time of writing, and complete results will be reported in a subsequent publication.

Preliminary qualitative data suggest that residents who participated in multiple sessions reported greater comfort discussing environmental health topics with patients and peers. Additionally, the curriculum generated interest in resident-led quality improvement projects addressing environmental determinants of health within the institution’s clinical settings.

### 4.4 Assessment framework

The evaluation strategy encompasses multiple levels of Kirkpatrick’s model of training evaluation (21). Level 1 (reaction) is assessed through post-session satisfaction and relevance ratings. Level 2 (learning) is measured through the pre-post confidence surveys and knowledge-based questions embedded in the post-curricular instrument. Level 3 (behavior) will be assessed in future phases through tracking of environmental health-related clinical documentation, patient counseling frequency, and advocacy activities. Level 4 (results) represents the long-term aspirational goal of improved patient outcomes related to environmental health counseling, which would require longitudinal follow-up beyond the scope of this initial implementation.

Each session’s learning objectives (Section 3.3) were mapped to specific items on the post-curricular survey to ensure alignment between intended outcomes and measured constructs. For example, the objective “identify patient populations susceptible to heat stress-induced kidney injury” from Session 1 corresponds to a confidence item on heat stress and kidney disease and a knowledge item on risk factor identification. This mapping ensures that the evaluation captures whether residents gained confidence and understanding in the specific domains targeted by each session.

## 5 Discussion

### 5.1 Practical implications

This curriculum represents, to our knowledge, one of the first published descriptions of a comprehensive, needs assessment-driven environmental health curriculum specifically designed for and implemented within an internal medicine residency program. While several groups have described climate and health education for pediatric residents (22–24) and proposed frameworks for residency education broadly (10), the internal medicine-specific literature has been limited to organ-system-based frameworks and single-institution reports (25). Our approach differs in its grounding in a published needs assessment that quantified specific confidence gaps across 12 environmental health domains, enabling targeted prioritization of curricular content.

The curriculum was designed with replicability as a guiding principle. By embedding sessions within existing protected didactic time, the intervention avoids the common barrier of competing for additional curricular hours—a challenge cited by over 71% of health professions school deans in a prior national survey (11, 26). The modular structure allows other programs to selectively adopt sessions most relevant to their regional environmental health profile. Programs in the Pacific Northwest might prioritize wildfire smoke and respiratory disease; programs in the Gulf South might emphasize hurricane-related displacement and infectious disease; programs in the Mountain West might focus on water scarcity and heat stress. The underlying pedagogical structure—needs assessment, self-efficacy-targeted objectives, expert-led interactive sessions, and pre-post confidence measurement—is transferable across these varied contexts.

### 5.2 Alignment with the emerging literature

Our findings are consistent with a rapidly growing body of evidence documenting the need for and feasibility of climate health education in GME. Moon et al. demonstrated that a single climate change session integrated into pediatric, family medicine, and social medicine curricula significantly improved residents’ self-reported knowledge and confidence (22). McShane et al. found that a 90-min interactive session on climate and health was well-received by pediatric residents and improved self-reported knowledge (23). Marwah et al. implemented a longitudinal four-part climate justice curriculum in a pediatrics program and found improvements in attitudes and advocacy intentions (24). Padgett et al. described a longitudinal climate and health curriculum for pediatric and internal medicine-pediatrics residents, finding low baseline comfort but evidence of feasibility (27).

What distinguishes our work is the focus on internal medicine as a standalone specialty, the breadth of environmental health topics assessed (12 domains rather than climate change alone), and the explicit grounding in a prior peer-reviewed needs assessment. The integration of environmental justice as a core theme—rather than an adjunct—reflects the reality that environmental health disparities are fundamentally equity issues, a perspective increasingly endorsed by the American College of Physicians and other professional organizations (8).

### 5.3 The role of self-efficacy in physician advocacy

The emphasis on self-efficacy in our curriculum design is supported by evidence linking trainee confidence to downstream clinical and advocacy behaviors. Studies have shown that physicians who report greater confidence in discussing environmental health with patients do so more frequently (28). Residents who feel comfortable discussing health topics with peers are more likely to describe advocacy as a core component of their professional identity (20). Lack of confidence in environmental health counseling among practicing clinicians has been traced to insufficient training during residency (29). Targeted confidence-building curricula in pediatrics have successfully increased both resident confidence and the frequency of environment-related patient counseling (30). These findings collectively suggest that interventions targeting self-efficacy, rather than knowledge alone, may produce more durable changes in clinical practice.

### 5.4 Lessons learned

Several practical insights emerged from the implementation process. First, engaging national and local stakeholders—including environmental health scientists, community organizations, and public health policy experts—enriched the curriculum’s content and lent credibility to what might otherwise be perceived as tangential to traditional internal medicine training. Second, interdisciplinary faculty recruitment was essential; the participation of a toxicologist from a national environmental organization and specialists in pulmonary medicine and infectious disease demonstrated to residents that environmental health is relevant across the spectrum of internal medicine. Third, the needs assessment was invaluable not only for prioritizing content but also for generating institutional buy-in, as the data provided an evidence base for allocating didactic time to this topic. Fourth, flexibility in scheduling and responsiveness to resident feedback during the implementation period allowed for real-time adjustments that improved engagement.

Despite these strengths, several challenges warrant candid reflection. Scheduling sessions during protected didactic time occasionally created conflicts with other educational priorities, requiring negotiation with subspecialty rotation directors and, in one instance, rescheduling a session to accommodate an unexpected clinical demand. Resident engagement, while generally positive, varied across sessions; attendance was highest for sessions on emerging infectious disease and air pollution—topics with immediate clinical applicability—and lower for sessions on endocrine disruptors and hazardous waste, which residents may have perceived as less directly relevant to inpatient practice. Faculty availability was an ongoing challenge, as content experts had competing clinical and research obligations; one session required a last-minute format adjustment when a presenter’s schedule changed and technical issues arose. Additionally, the absence of longitudinal reinforcement between sessions meant that content from earlier modules may have been insufficiently retained by the time later sessions were delivered.

A particularly notable observation was that PGY-1 residents reported higher baseline confidence in environmental justice and toxicology than their senior peers. This may reflect the increasing incorporation of social determinants of health and planetary health content into undergraduate medical education (19), suggesting a generational shift that residency programs should be prepared to build upon rather than duplicate.

In future iterations, we would make several modifications. First, we would add brief refresher activities (e.g., a 5-min case vignette reviewing the prior session’s key concepts) at the start of each new session to promote retention and continuity. Second, we would develop a standardized facilitator guide with more prescriptive timing and discussion prompts to reduce variability across presenters. Third, we would incorporate a matched pre-post knowledge, rather than confidence, assessment to complement the confidence measures. Fourth, we would explore integration of environmental health content into clinical rotations—such as prompting environmental exposure history-taking during outpatient continuity clinic—to reinforce didactic learning with practice-based application.

## 6 Acknowledgment of constraints

Several conceptual, methodological, and practical constraints must be acknowledged. First, this curriculum was developed and implemented at a single institution within a single specialty, limiting generalizability. The environmental health priorities of central Texas may not be directly transferable to other regions, though the underlying pedagogical framework is designed to be adaptable.

Second, the needs assessment response rate (56%), while reasonable for survey research in residency populations, did not achieve a fully representative sample (margin of error 8.26%). Respondents may have been disproportionately motivated by personal interest in environmental health, potentially inflating or deflating confidence estimates.

Third, the use of self-reported confidence as the primary outcome measure has inherent limitations. Confidence is not equivalent to competence, and the relationship between self-efficacy and actual clinical performance, while theoretically supported, is not always linear (14). Moreover, the current evaluation design does not include a validated, pre-post knowledge assessment, meaning that changes in factual understanding cannot be rigorously measured alongside changes in confidence. This represents an important limitation, as confidence without corresponding knowledge growth could reflect overestimation of competence rather than genuine skill development. Future iterations should incorporate objective knowledge assessments, observed clinical encounters, and behavioral measures such as environmental exposure history documentation rates.

Fourth, the post-curricular assessment data collection is ongoing, and complete pre-post results are not available for this publication. A subsequent analysis will report these findings in detail.

Fifth, some survey items were double-barreled (e.g., “food security and sustainability”), introducing ambiguity about which sub-concept respondents were evaluating. Additionally, the survey instrument was developed *de novo* and lacks established validity evidence beyond its grounding in prior self-efficacy scales (12).

Finally, the curriculum was delivered during a period of significant national attention to climate change and environmental policy, which may have independently influenced resident attitudes and engagement. Isolating the curricular effect from broader cultural influences is not possible in this study design.

## 7 Future directions

Future work will focus on several priorities. First, completion of the post-curricular confidence assessment will allow formal pre-post comparison and identification of domains with the greatest improvement. Second, longitudinal tracking of residents’ environmental health counseling behaviors—through chart review, patient surveys, or standardized patient encounters—will provide higher-level evaluation data. Third, expansion of the curriculum to include simulation-based exercises (e.g., standardized patient encounters involving environmental exposure histories) would provide mastery experiences more closely aligned with Bandura’s theory. Fourth, multi-institutional collaboration to replicate and evaluate this curricular model across diverse geographic and programmatic settings would strengthen the evidence base and support broader adoption. Fifth, exploration of digital resources, such as online modules or virtual case libraries, could extend the reach of environmental health education beyond individual residency programs.

As the health consequences of climate change continue to accelerate, the imperative to prepare the physician workforce grows correspondingly urgent. Internal medicine residents, who will provide comprehensive care to adult patients across the lifespan and across clinical settings, must be equipped with the knowledge, confidence, and advocacy skills to address environmental determinants of health. The curriculum described here offers one model for answering this call.

## Data Availability

The data analyzed in this study is subject to the following licenses/restrictions: previously analyzed data set that is no longer available. Requests to access these datasets should be directed to jessica.chambers@austin.utexas.edu.
